# Comparative transcriptome analysis uncovers regulatory roles of long non-coding RNAs involved in resistance to powdery mildew in melon

**DOI:** 10.1186/s12864-020-6546-8

**Published:** 2020-02-05

**Authors:** Chao Gao, Jianlei Sun, Yumei Dong, Chongqi Wang, Shouhua Xiao, Longfei Mo, Zigao Jiao

**Affiliations:** 1 Shandong Key Laboratory of Greenhouse Vegetable Biology, Shandong Branch of National Improvement Center for Vegetable, Vegetable Science Observation and Experiment Station in Huang huai District of Ministry of Agriculture (Shandong), Institute of Vegetables and Flowers, Shandong Academy of Agricultural Sciences, Jinan, 250100 China; 2 College of horticulture, Jilin Agricultural University, Changchun, 130118 China

**Keywords:** Melon, Comparative transcriptome, Long non-coding RNA, Powdery mildew disease, Expression pattern

## Abstract

**Background:**

Long non-coding RNAs (lncRNAs) are a class of non-coding RNAs with more than 200 nucleotides in length, which play vital roles in a wide range of biological processes. Powdery mildew disease (PM) has become a major threat to the production of melon. To investigate the potential roles of lncRNAs in resisting to PM in melon, it is necessary to identify lncRNAs and uncover their molecular functions. In this study, we compared the lncRNAs between a resistant and a susceptible melon in response to PM infection.

**Results:**

It is reported that 11,612 lncRNAs were discovered, which were distributed across all 12 melon chromosomes, and > 85% were from intergenic regions. The melon lncRNAs have shorter transcript lengths and fewer exon numbers than protein-coding genes. In addition, a total of 407 and 611 lncRNAs were found to be differentially expressed after PM infection in PM-susceptible and PM-resistant melons, respectively. Furthermore, 1232 putative targets of differently expressed lncRNAs (DELs) were discovered and gene ontology enrichment (GO) analysis showed that these target genes were mainly enriched in stress-related terms. Consequently, co-expression patterns between LNC_018800 and *CmWRKY21*, LNC_018062 and *MELO3C015771* (glutathione reductase coding gene), LNC_014937 and *CmMLO5* were confirmed by qRT-PCR. Moreover, we also identified 24 lncRNAs that act as microRNA (miRNA) precursors, 43 lncRNAs as potential targets of 22 miRNA families and 13 lncRNAs as endogenous target mimics (eTMs) for 11 miRNAs.

**Conclusion:**

This study shows the first characterization of lncRNAs involved in PM resistance in melon and provides a starting point for further investigation into the functions and regulatory mechanisms of lncRNAs in the resistance to PM.

## Background

It has been reported that a large portion of the genomic sequences is transcribed [1]. However, only few transcripts encode protein sequences in eukaryotic organisms, suggesting that most transcripts are non-coding RNA (ncRNA) [2]. The ncRNA families are composed of small and long non-coding RNA (lncRNAs) based on the length of mature transcripts. Small ncRNAs (approximately 18–30 nucleotides) include microRNAs (miRNAs) and small interfering RNAs (siRNAs), which have been well characterized for their involvement in the regulation of gene expression at transcriptional and post-transcriptional level in almost all eukaryotes [3]. LncRNAs are a class of non-coding RNAs with more than 200 nucleotides in length, which have been demonstrated to participate in the regulation of gene expression during plant growth and development, and various stress responses of plants [4–6]. According to their position on the genome, lncRNAs can be classified into long intergenic non-coding RNA (lincRNA), long intronic non-coding RNAs and natural antisense transcripts (lncNATs) [7].

Over the last decades, with the development of high-throughput sequencing, thousands of lncRNAs have been identified in various plant species, such as *Arabidopsis*, rice, maize, tomato, apple, strawberry and others [8–13]. Many lncRNAs have been functionally characterized in some plants, especially in *Arabidopsis* and rice, indicating that lncRNAs play critical roles in multiple biological processes including flowering, photomorphogenesis, sex differentiation, and fruit development [14]. In Arabidopsis, 6480 transcripts have been classified as lncRNAs. Among them, one intronic lncRNA transcribed from the first intron of *FLOWERING LOCUS C* (*FLC*) and two antisense lncRNAs transcribed from the antisense strand of *FLC* have been reported to affect the flowing time by negatively regulating *FLC* expression at epigenetic and post-transcriptional level after cold condition [15]. In rice, it was found that lncRNAs expressed in highly tissue-specific or stage-specific manner, and a set of lncRNAs have been demonstrated to be involved in photoperiod-sensitive male sterility and sexual reproduction [16]. In tomato, 490 lncRNAs were significantly up-regulated in ripening mutant fruits *rin*, and 187 lncRNAs were down-regulated, implying that lncRNAs could be involved in the regulation of fruit ripening in tomato [13]. Indeed, silencing of two intergenic lncRNAs in wild-type fruit (lncRNA1459 and lncRNA1840) resulted in an obvious delay of fruit ripening [13].

LncRNAs are also responsive to various biotic and abiotic stresses, and have been confirmed to play significant roles in several biological processes of plant stress responses, such as drought, salt stress and various pathogen stresses [17, 18]. *Drought induced lncRNA* (*DRIR*) in *Arabidopsis* was expressed at a low level after non-stress conditions but can be significantly activated by drought, salt stress and abscisic acid treatment, which contributes to salt and drought tolerance [19]. In plant-pathogen interactions, lncRNAs also played vital roles in plant’s defense system during pathogen infection [20]. In tomato, it was found that slylnc0195 acted as competing endogenous target mimics for miR166 to protect its targets, class III HD-Zip transcription factor genes, and was involved in the resistance against TYLCV infection [18]. Moreover, a set of *F. oxysporum*-induced lncRNAs (15 lncNATs and 20 lincRNAs) were identified in *Arabidopsis*, and the role of lincRNAs for resistance against *F. oxysporum* was functionally confirmed using T-DNA insertion or RNA-interference knockdown lines [17]. Furthermore, promoter analysis suggested that some of the *F. oxysporum*-induced lncTARs were direct targets of transcription factors responsive to pathogen attack [17]. Collectively, these studies showed that lncRNAs play important roles during plant growth and development as well as in resisting to various stresses. However, research has not been reported in melon, and little is known about lncRNAs and their potential roles in melon.

Melon (*Cucumis melo* L.) is an economically important fruit crop that belongs to *Cucurbitaceae* family, and is susceptible to powdery mildew disease (PM) during the later stage of development [21]. PM is a kind of fungal disease of melon caused by *Podosphaera xanthii* (Px) or *Golovinomyces cichoracearum* (Gc), which leads to the decline of melon yield and quality, and PM has severely hindered the development of melon industry [21]. To identify lncRNAs in melon and assess their potential roles in resisting to PM, we used comparative whole transcriptome analysis of PM-resistant and PM-susceptible melon leaves after PM inoculation to identify differentially expressed lncRNAs and investigate lncRNA-mRNA networks. Our results indicated that a large number of lncRNAs were responsive to PM infection, including those that act as endogenous miRNA target or mimics (eTMs), which provided a foundation for further functional analysis of lncRNAs in the resistance to PM.

## Results

### Different phenotype of M1 and B29 after powdery mildew infection

**Fig. 1:**
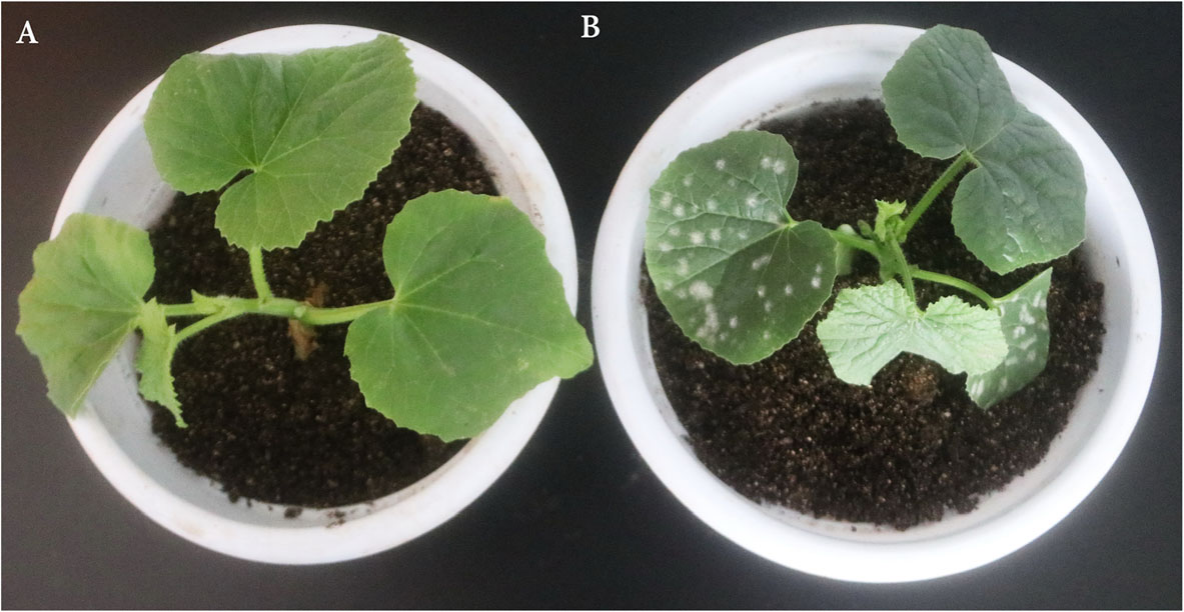
Different phenotype of two melons observed at 7 day after powdery mildew infection. **a**: the phenotype of M1; **b**: the phenotype of B29

The occurrence of PM disease was assessed after inoculation with powdery mildew fungus in the greenhouse. As shown in Fig. 1a, no obvious bacterial plaque was observed on M1 leaves at 7 day after powdery mildew infection, while the B29 leaves were wisped with intense mildew (Fig. 1b), indicating the significant difference in resisting to PM between the two genotypes. Previous transcriptome profiling analysis of genes in melon after PM inoculation revealed that the expression of genes involved in the response to biotic stimulus resistance, response to external stimuli, signal transduction, kinase activity, transcription factor activity and plant-pathogen interactions was increased at 24 hpi and high expression levels were maintained to 48 hpi, and was subsequently decreased after 48 hpi [22]. Given that the disease resistance response in melon generally occurred before phenotype observed, leaves of both M1 and B29 genotypes were harvested at 24, 48 h post inoculation for further analysis.

### Overview of RNA-seq data

High-throughput sequencing was performed to identify lncRNAs and evaluate their expression in the leaves of PM-resistant lines (M1) and PM-susceptible lines (B29) infected at 0, 24 and 48 hpi. In this study, three biological replicates were used and a total of 18 libraries were sequenced in a 150 bp paired-end module. In all samples, approximately 82.68 to 85.97% of clean reads were uniquely mapped to the melon reference genome. The rates of genomic match were similar among different samples, suggesting the similar quality of sequence data across the series. Detailed mapping statistics is provided in Additional file 1: Table S1. Based on the expression value of FPKM, correlation coefficient of three biological replicates for each sample was calculated. The correlation coefficients were > 0.94 for almost all comparisons, suggesting that there was a perfect correlation among the biological replicates (Additional file 2: Figure S1).

### Whole-transcriptome identification and characterization of lncRNAs in melon

**Fig. 2:**
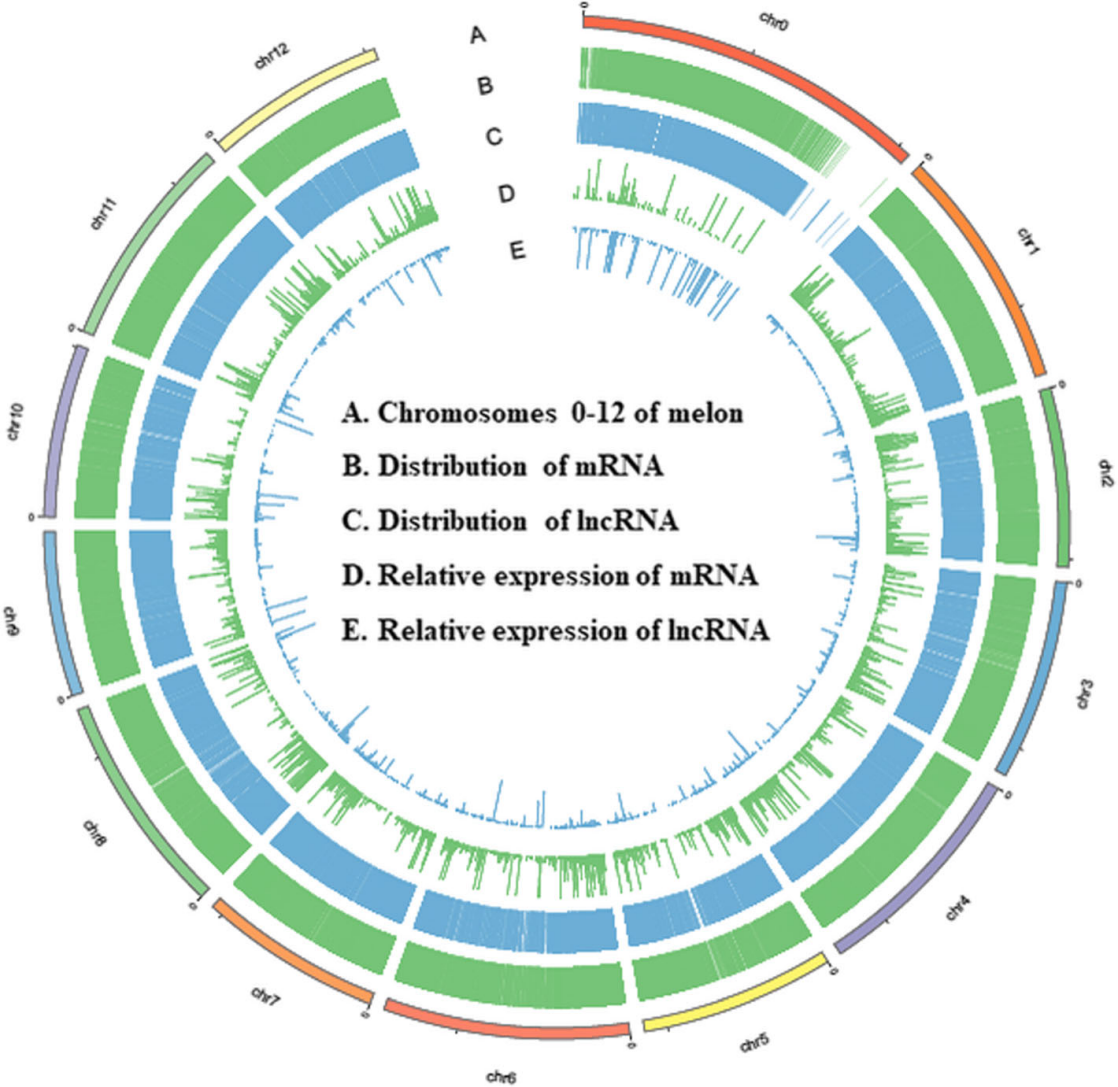
Genome-wide distribution and expression of melon lncRNAs compared to that of protein-coding mRNAs. The expression level of lncRNAs and protein-coding mRNAs is presented as Log_10_FPKM

**Fig. 3:**
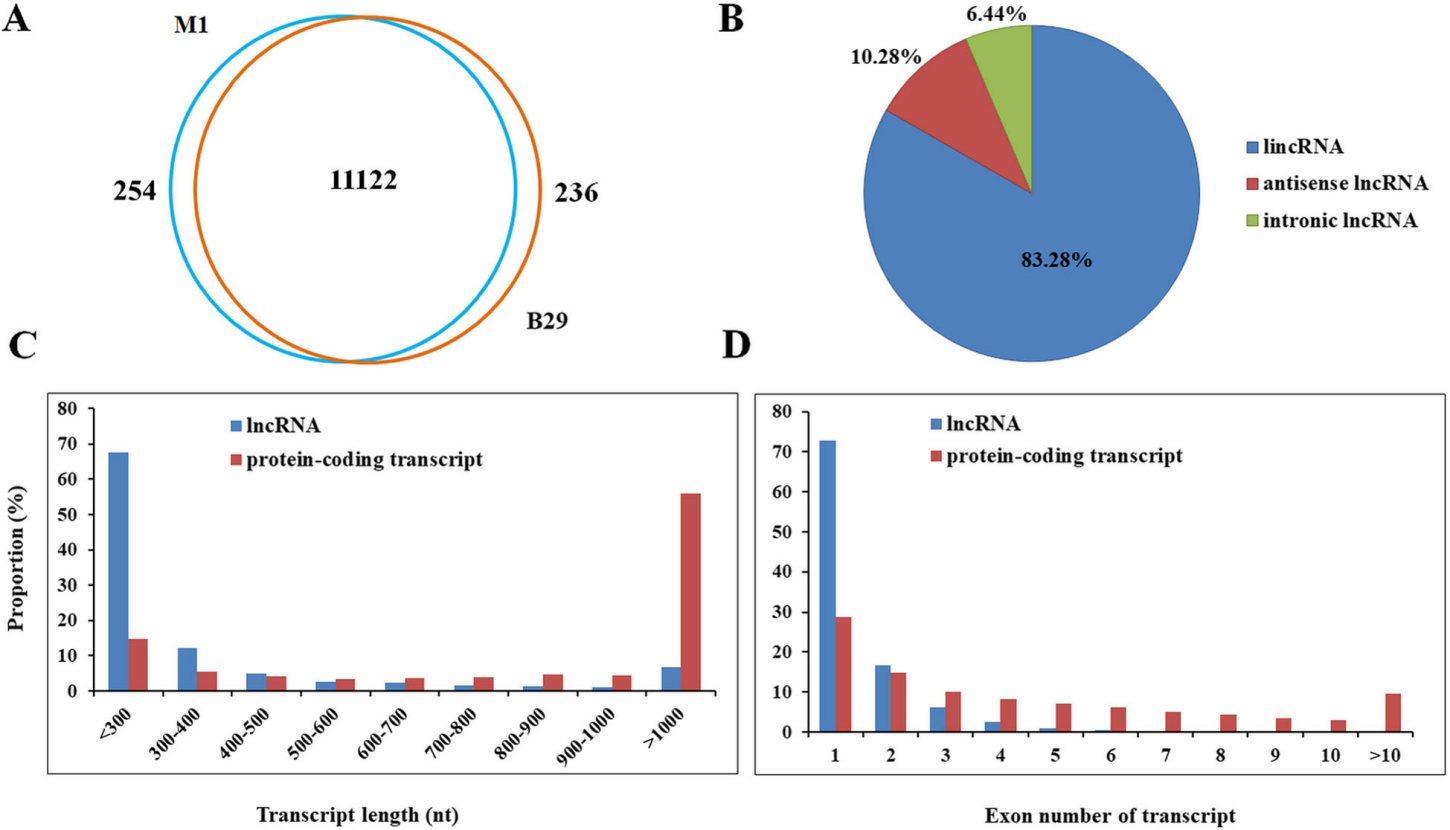
Identification and characterization of lncRNAs in PM-susceptible and PM-resistant melons. **a**. Number of shared and specific lncRNAs between B29 and M1. **b**. Classification of melon lncRNAs according to its genomic position. **c**. The distribution of length of all lncRNAs identified in melon. **d**. The distribution of exon number of lncRNAs identified in melon

A total of 124,979 unique transcripts were obtained from RNA-Seq data merged from all 18 samples. After seven sequential stringent filters (see materials and methods), 11,612 lncRNAs were identified, which were evenly distributed across 12 chromosomes in melon (Fig. 2). Among them, 11,122 lncRNAs were accumulated in both M1 and B29, and only 254 and 236 unique lncRNAs were specifically expressed in M1 and B29, respectively (Fig. 3a). Based on their genomic location and orientation relative to the nearest protein coding genes, lncRNAs are classified into lincRNA, intronic lncRNA and antisense lncRNA. Approximately 83.28% lncRNAs belonged to lincRNAs, 10.28% lncRNAs belonged to antisense lncRNA, and 6.44% lncRNAs were classified into intronic lncRNA in melon (Fig. 3b). The length and exon number of melon lncRNAs were analyzed compared with protein-coding transcripts for their characterization. As shown in Fig. 3c, the length of most lncRNAs (~ 68%) ranged within 200–300 nucleotides, whereas the length of most protein-coding transcripts mainly ranged in the size of > 1000 nucleotides in melon. In addition, majority lncRNAs (90%) contained one or two exons, while the number of exons for protein-coding genes ranged from one to ≥10 (Fig. 3d). These results indicated that the majority of melon lncRNAs were relatively shorter in length and contained fewer exons compare to protein-coding transcripts.

### Differential expression of lncRNAs in response to PM infection

**Fig. 4:**
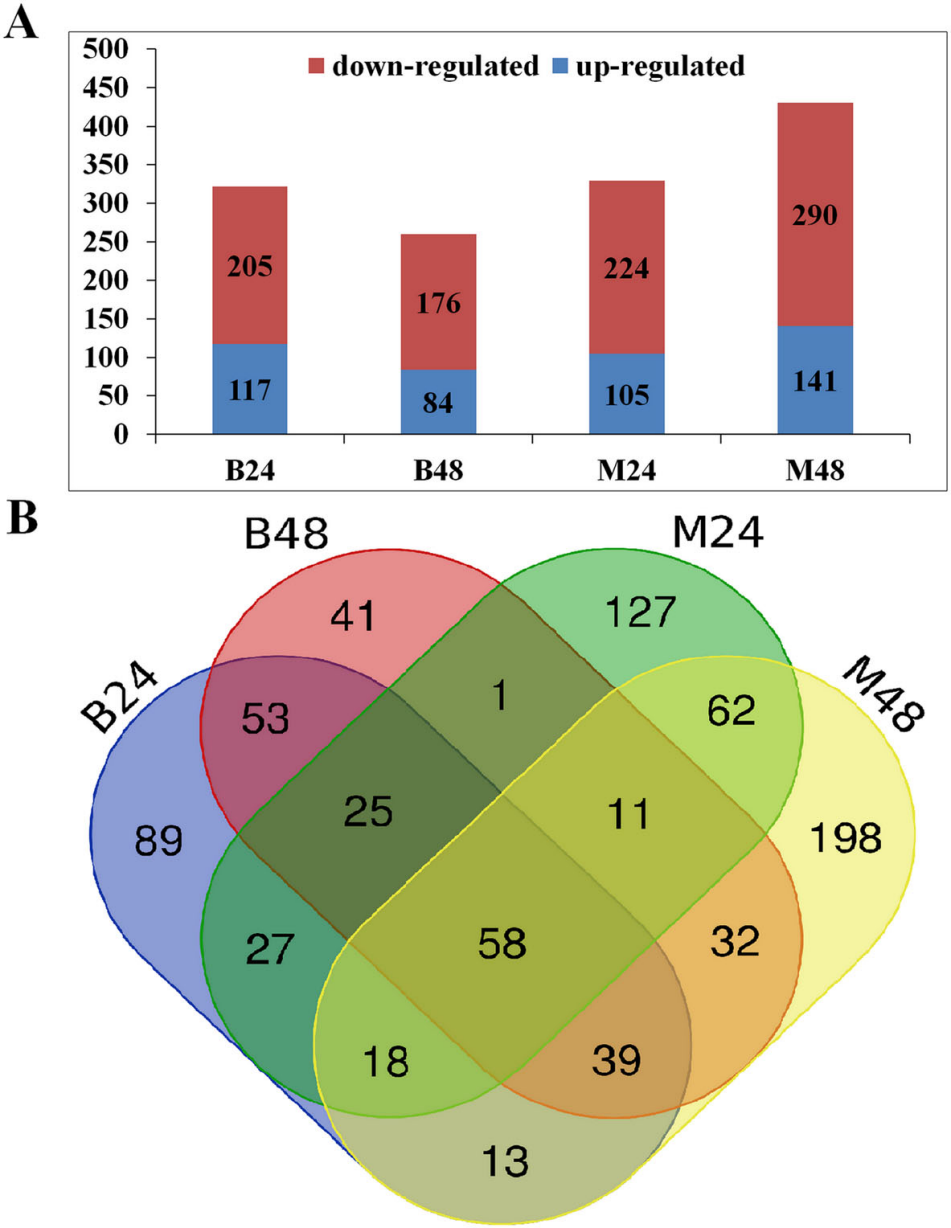
Statistical analysis of DELs between PM-susceptible melon (B29) and PM-resistant melon (M1). **a**. Number of down- and up-regulated lncRNAs at 24 and 48 hpi compared with mock in B29 and M1. **b**. Number of shared and specific DELs in B29 and M1

**Fig. 5:**
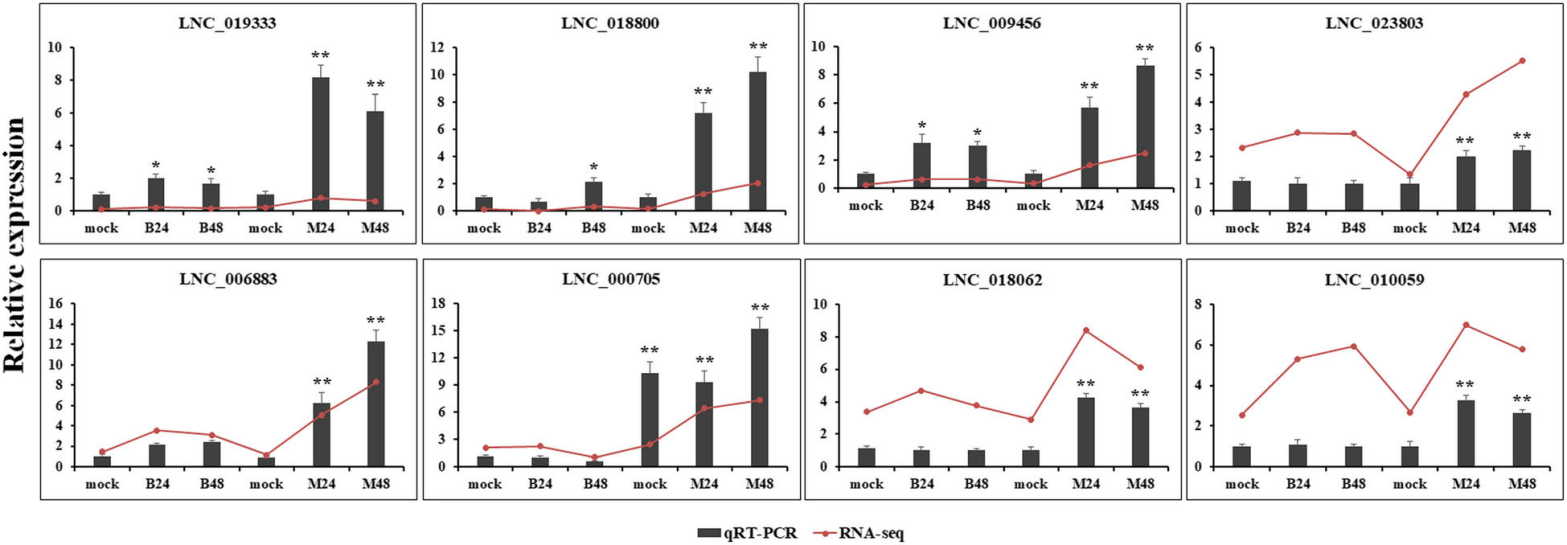
Experimental validation of eight highly altered DELs by qRT-PCR. *CmActin* was used as internal reference. Relative level of lncRNAs was normalized to that in mock. The RNA-seq values were presented as log2 (FPKM value + 1). Error bars indicate±SD of three biological replicates. Asterisks indicated a significant change (*P < 0.05; **P < 0.01)

To identify PM-responsive lncRNAs, their differential expressions were evaluated between PM infected samples and mock samples for both PM-resistant and PM-susceptible melons. The lncRNAs expressed with |log_2_ fold change| ≥ 1 and adjusted *P*-values < 0.01 were designated as DELs. More DELs were identified in PM-resistant melon compared to PM-susceptible melon, while the number of down-regulated DELs was greater than that of up-regulated DELs in all comparison groups. As a result, a total of 117, 84, 105, 141 lncRNAs were found to be significantly up-regulated in B24, B48, M24, M48, respectively. Furthermore, a total of 205, 176, 224, 290 lncRNAs were found to be significantly down-regulated in B24, B48, M24, M48, respectively (Fig. 4a). Additionally, a total of 183 nd 387 lncRNAs were specifically differentially expressed in PM-susceptible melon and PM-resistant melon, respectively (Fig. 4b). The differential expression levels of eight highly altered DELs were experimentally validated by qRT-PCR. The results showed that the expression of LNC_010059, LNC_018602, LNC_023803 were significantly up-regulated at 24 and 48 hpi in PM-resistant melon after PM infection. However, the expression levels of these three lncRNAs were not changed in PM-susceptible melon (Fig. 5). Moreover, qRT-PCR analysis confirmed that the accumulation of LNC_000705, LNC_006883, LNC_009456, LNC_018800, LNC_019333 in PM-resistant melon were highly induced than that in PM-susceptible melon after PM infection, which were consistent with the RNA-seq results (Fig. 5), suggesting that the high throughput data were reliable.

### Target prediction and functional characterization of differentially expressed lncRNAs

**Fig. 6:**
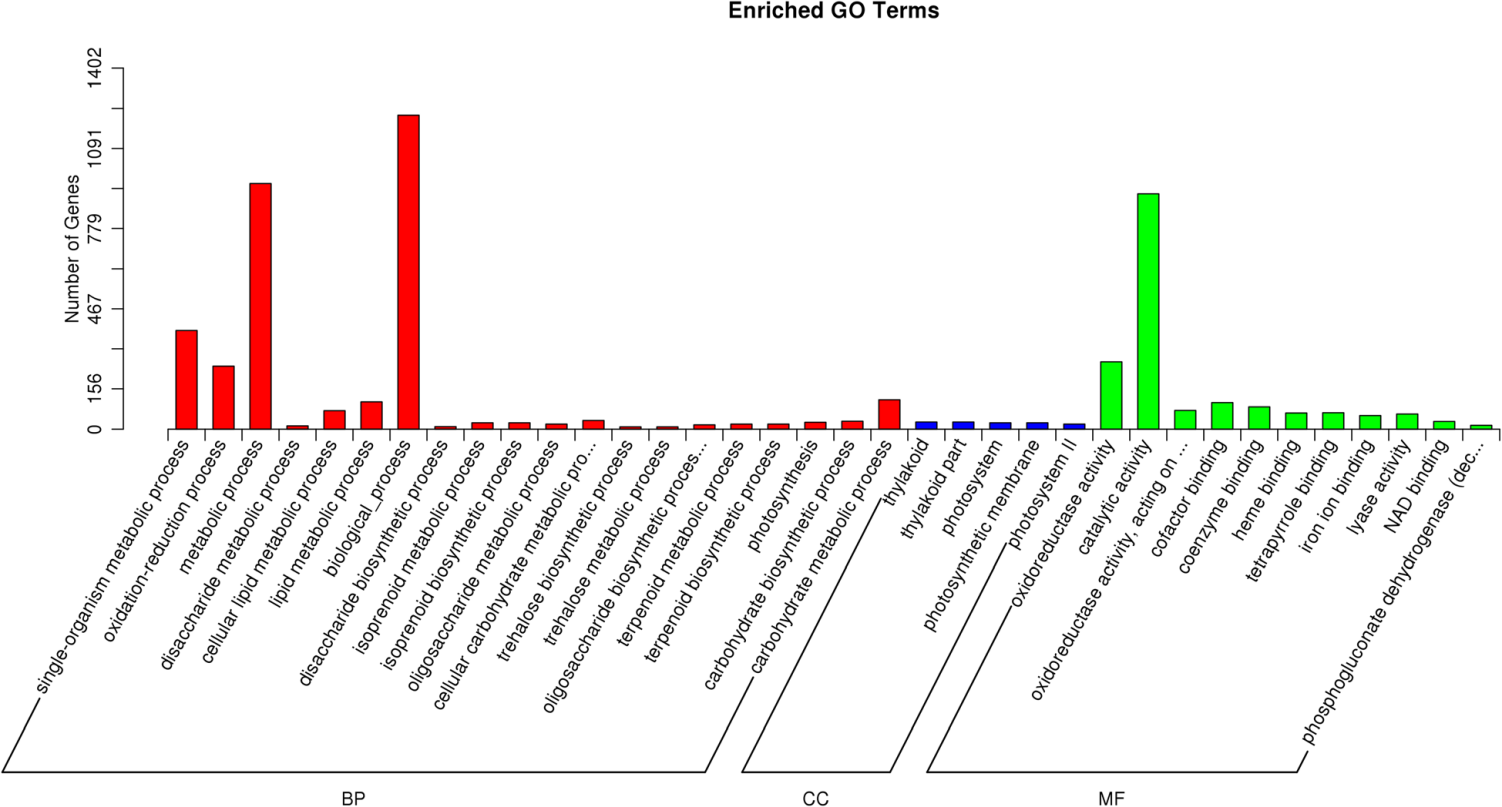
GO annotation and enrichment analysis for the target genes of DELs. Go terms distribution of target genes under molecular functions, cellular components, and biological processes

Generally, lncRNAs function in controlling the expression of their *cis*- or *trans*-target genes by forming lncRNA-target duplexes. In order to reveal the potential functions and regulatory mechanism of lncRNAs in response to PM infection, we characterized the target genes that were located < 10 kb from the DELs and analyzed their Gene Ontology (GO) terms. A total of 1232 protein-coding genes were predicted as target genes for all DELs, and these target genes were mainly enriched in three main GO categories, such as cellular component, molecular function and biological process (Fig. 6). The most abundant GO terms in the biological process were cell activation involved in immune response (GO: 0002263), metabolic process (GO: 0006629, lipid metabolic process), oxidation-reduction process (GO: 0004601, peroxidase activity; GO: 0045454, cell redox homeostasis) (Additional file 3: Figure S2). In addition, the molecular functions of these target genes were mainly enriched in “catalytic activity” and “oxidoreductase activity” (Fig. 6). The enrichment result suggested that the differentially expressed lncRNAs after PM infection may regulate the protein-coding genes involved in several important biological processes to resisting to PM infection.

### Identification of PM-resistant genes and expression analysis after PM infection

**Fig. 7:**
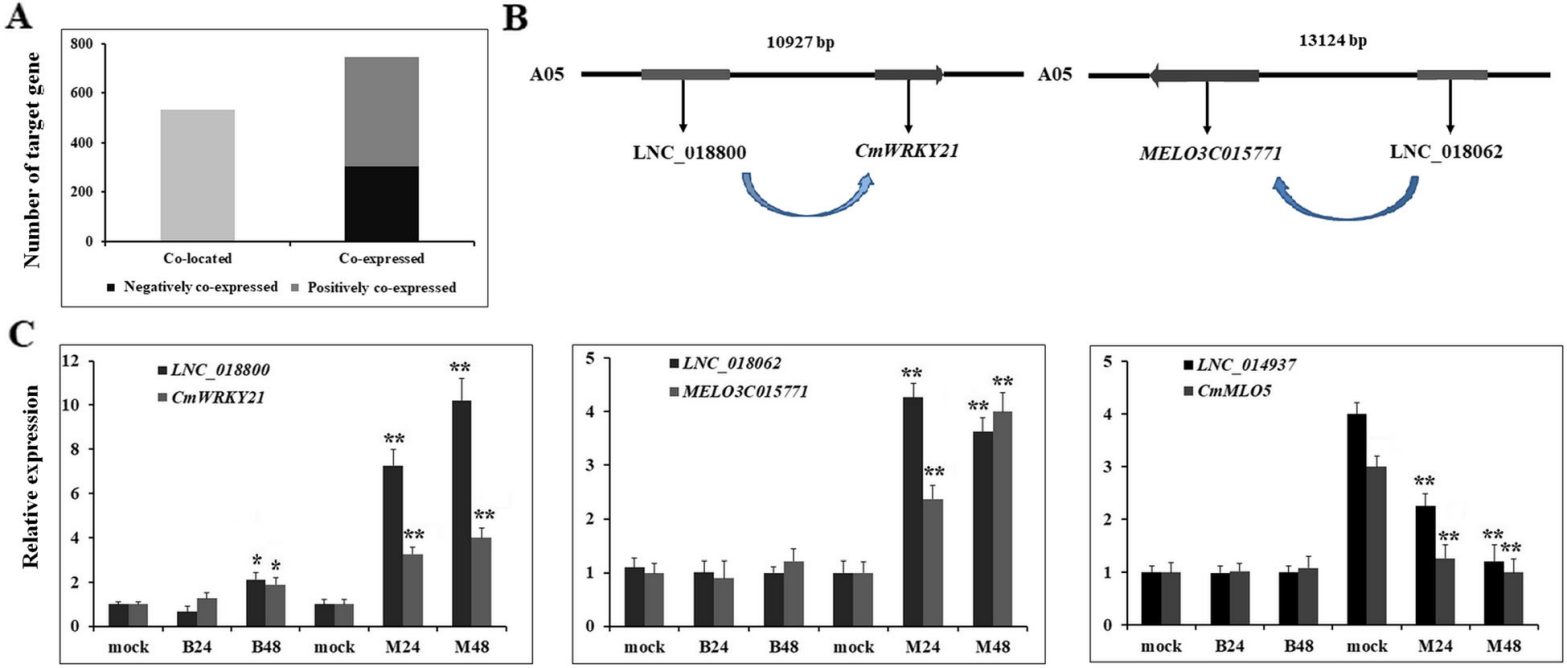
Location of two PM-responsive lncRNAs with their target genes and validation of their differential expression after PM infection by qRT-PCR. **a**. The number statistics of target genes of 387 DELs that were specific to PM-resistant melon. **b**. Gene structures of two lncRNAs and their neighboring protein-coding genes. **c**. Experimental validation of the expression patterns of lncRNAs and their target genes. *CmActin* was used as internal reference. Relative expression level of lncRNAs and target genes was normalized to that in mock. Error bars indicate±SD of three biological replicates. Asterisks indicated a significant change (*P < 0.05; **P < 0.01)

**Fig. 8:**
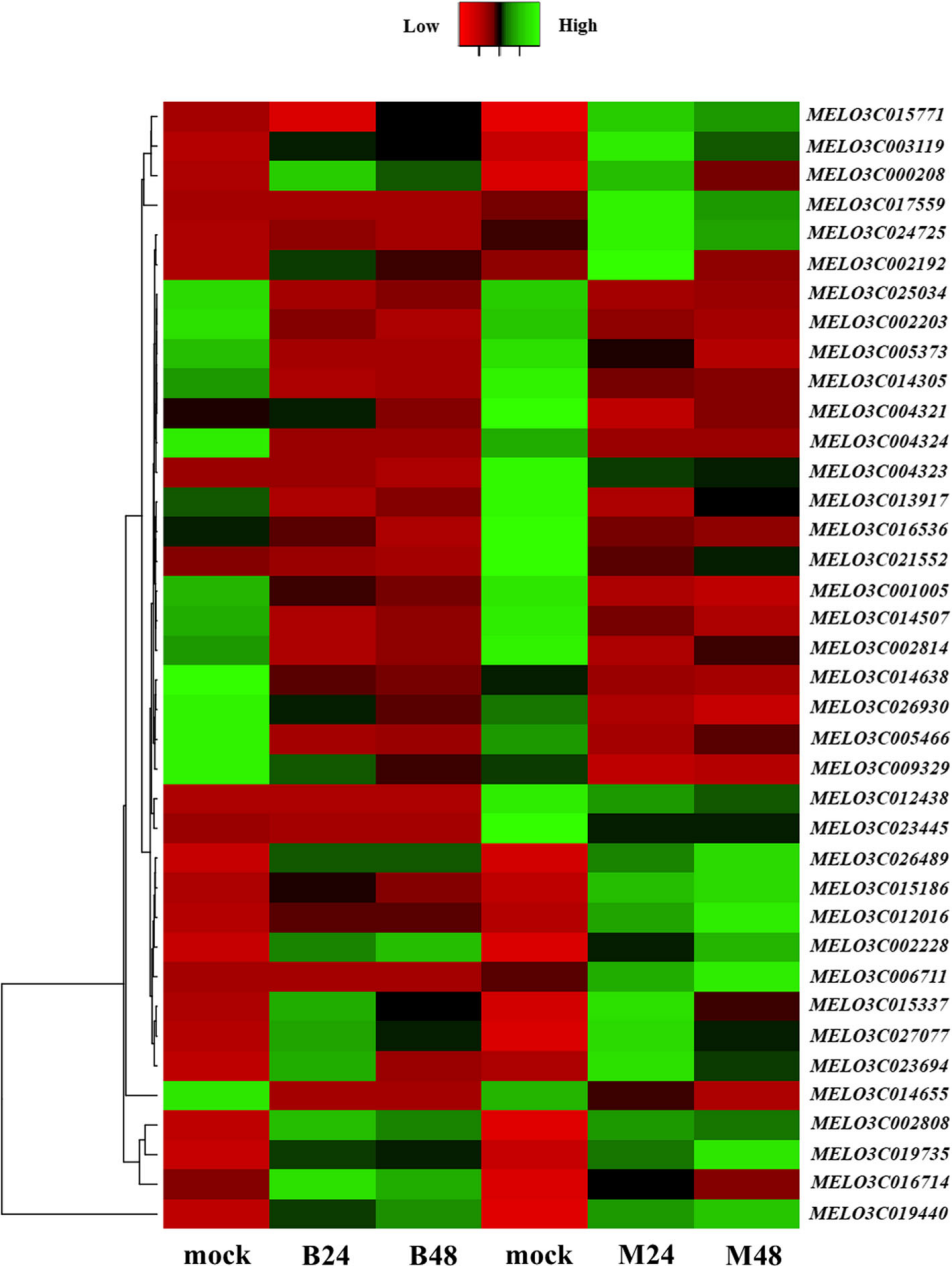
Differential expression patterns of disease-related target genes of DELs after PM infection. The expression values were measured as fragments per kilobase of exon model per million mapped reads (FPKM) and presented as log2 (value + 1). High expression levels are shown in green and low expression levels are shown in red

With further analysis of the target genes of 387 DELs that were specific to PM-resistant melon, it was found that 532 protein-coding genes were co-located with DELs, and 440 and 335 protein-coding genes were positively co-expressed and negatively co-expressed with those DELs, respectively (Fig. 7a). Among those target genes, eight genes that might be directly involved in disease resistance were co-located with five DELs, and 30 genes that might be involved in PM resistance were co-expressed with 27 DELs (Table 1). *MELO3C002814*, encoding a LRR receptor-like kinase, was found to be located in the downstream 14,128 bp of LNC_010059 (Fig. 7b). Similarly, *MELO3C014305*, encoding a WRKY transcription factor, was found to be located in the upstream 10,972 bp of LNC_018800 (Fig. 7b). Furthermore, *MELO3C015771*, encoding a glutathione reductase, was co-expressed with LNC_018062 with a correlation coefficient of 0.96. To validate the putative expression patterns between DELs and their target genes, the expression levels of three DELs and their target genes after PM inoculation in both PM-susceptible and PM-resistant melon were examined by qRT-PCR. It was found that *CmWRKY21* and its paired lncRNA (LNC_018800), LNC_018062 and its paired target gene (*MELO3C015771*) exhibited a similar pattern in both PM-resistant melon and PM-susceptible melon, with up-regulated after PM infection in PM-resistant melon (Fig. 7c). Meanwhile, LNC_014937 and its paired target gene (*CmMLO5*) showed a similar decreased pattern in PM-resistant melons (Fig. 7c). In addition, the expression patterns of 38 PM-resistant genes are shown in a heatmap (Fig. 8). In particular, it was found that the accumulation levels of *MELO3C023445*, *MELO3C006711*, *MELO3C017559*, *MELO3C024725* and *MELO3C004323* in PM-resistant melon were much higher than that in PM-susceptible melon (Fig. 8). More importantly, these genes were significantly up- or down-regulated in PM-resistant melon at both 24 and 48hpi and no obvious differential expression of those genes was found in PM-susceptible melon after PM infection (Fig. 8). In addition, the expression of *MELO3C012438* that encodes a Mildew Locus O (MLO) protein was decreased in PM-resistant melon after PM infection and no differential expression was observed in PM-susceptible melon.

**Table 1:** Identification of target genes of DELs that related to diease resistance in melon

LncRNA ID	Target gene ID	Functional annotation	Relationship
LNC_010059	MELO3C002814	LRR receptor kinase	Co-located
LNC_010059	MELO3C002808	GTPase-activating protein	Co-located
LNC_000705	MELO3C001005	Cytochrome P450	Co-located
LNC_023803	MELO3C017559	Cytochrome P450	Co-located
LNC_019333	MELO3C004321	Disease resistance protein	Co-located
LNC_019333	MELO3C004323	Disease resistance protein	Co-located
LNC_019333	MELO3C004324	Disease resistance protein	Co-located
LNC_018800	MELO3C014305	WRKY21	Co-located
LNC_009456	MELO3C024725	Disease resistance protein	Co-expressed
LNC_006883	MELO3C012016	MLP-like protein	Co-expressed
LNC_006883	MELO3C015337	NADPH-dependent reductase	Co-expressed
LNC_018062	MELO3C015771	Glutathione reductase	Co-expressed
LNC_008020	MELO3C014655	Peroxidase	Co-expressed
LNC_018763	MELO3C019440	Ferredoxin	Co-expressed
LNC_016838	MELO3C023445	LRR domain protein	Co-expressed
LNC_014937	MELO3C012438	MLO	Co-expressed
LNC_012950	MELO3C021552	Universal stress protein	Co-expressed
LNC_003246	MELO3C016714	Protochlorophyllide reductase	Co-expressed
LNC_009567	MELO3C019735	ACO	Co-expressed
LNC_020345	MELO3C016536	NAC	Co-expressed
LNC_006685	MELO3C013917	ERF5	Co-expressed
LNC_016838	MELO3C014507	F-box protein	Co-expressed
LNC_003521	MELO3C005466	ERF	Co-expressed
LNC_017611	MELO3C009329	Peroxidase	Co-expressed
LNC_021129	MELO3C026930	ABA receptor	Co-expressed
LNC_018663	MELO3C014638	Lipoxygenase	Co-expressed
LNC_008020	MELO3C005373	F-box protein	Co-expressed
LNC_006033	MELO3C015186	ferredoxin	Co-expressed
LNC_012422	MELO3C002203	aldehyde dehydrogenase	Co-expressed
LNC_025657	MELO3C025034	Peroxiredoxin	Co-expressed
LNC_003761	MELO3C003119	Heat shock protein	Co-expressed
LNC_011956	MELO3C006711	Universal stress protein	Co-expressed
LNC_002899	MELO3C002192	Cytochrome P450	Co-expressed
LNC_011592	MELO3C026489	Cytochrome P450	Co-expressed
LNC_011422	MELO3C000208	Heat shock protein	Co-expressed
LNC_010270	MELO3C002228	MYB1R1	Co-expressed
LNC_025117	MELO3C023694	Pathogen-related protein	Co-expressed
LNC_000319	MELO3C027077	Heat shock protein	Co-expressed

### LncRNA act as precursors, targets or eTMs of miRNAs

Numerous studies have reported that lncRNAs can interact with other ncRNAs such as miRNA to regulate various biological processes in many plants [23, 24]. On the one hand, many lncRNAs can act as potential miRNA precursors. On the other hand, lncRNAs could be targeted by miRNAs. In addition, plant lncRNAs could act as eTMs by binding to specific miRNA, competing with the target mRNA of miRNA and thus blocking the cleavage and alleviating the repression of its target gene [23]. In the present study, 23 lncRNAs were identified as the precursors of 19 miRNA families, including miR160, miR319, miR394, miR398 and miR408 that have been reported to play significant roles in mediating plant responses to phytopathogens (Table 2). Meanwhile, 43 lncRNAs were predicted as the potential targets of 22 miRNA families and 13 lncRNAs as eTMs of 11 miRNAs (Table 3). For a fraction of miRNAs, only one target was identified, such as miR162, miR319, miR390 and others. However, most miRNAs were found to have more than one target genes. For example, six lncRNAs and seven lncRNAs were targeted by miR156 and miR7129, respectively. Among these miRNA targets, LNC_014183 was the target of miR398 that was recently confirmed to be involved in plant immunity.

**Table 2:** LncRNAs acting as miRNA precusors in melon

lncRNA ID	lncRNA start	lncRNA end	miRNA ID	pre-miRNA length	Identity	*E*-value
LNC_013051	236	417	Cm-miR156	182	97	2.00E-27
LNC_011277	323	532	Cm-miR159	210	95	4.00E-28
LNC_006685	464	562	Cm-miR160	99	93	3.00E-31
LNC_012140	515	617	Cm-miR162	103	95	2.00E-32
LNC_026045	112	202	Cm-miR164a	91	100	2.00E-35
LNC_014811	593	681	Cm-miR164b	89	95	4.00E-30
LNC_001873	220	369	Cm-miR166	150	97	3.00E-31
LNC_004282	359	518	Cm-miR167a	160	94	4.00E-29
LNC_020891	51	156	Cm-miR167c	106	93	2.00E-29
LNC_010843	105	308	Cm-miR167d	204	92	2.00E-28
LNC_017947	119	235	Cm-miR167e	117	93	4.00E-32
LNC_023691	106	315	Cm-miR168	210	92	4.00E-30
LNC_007808	245	380	Cm-miR171	136	97	1.00E-28
LNC_000014	497	602	Cm-miR172	106	95	1.00E-30
LNC_007125	236	475	Cm-miR319	240	91	4.00E-30
LNC_022969	192	337	Cm-miR394	146	94	2.00E-30
LNC_006844	55	143	Cm-miR395	89	97	7.00E-34
LNC_002209	60	182	Cm-miR396a	123	96	1.00E-29
LNC_011916	132	282	Cm-miR396b	151	95	1.00E-31
LNC_010188	173	265	Cm-miR397	93	95	2.00E-36
LNC_011679	115	214	Cm-miR398	100	100	2.00E-29
LNC_006969	1228	1361	Cm-miR408	134	97	4.00E-29
LNC_020158	73	181	Cm-miR477	109	97	7.00E-26
LNC_007291	157	320	Cm-miR858	164	90	1.00E-28

**Table 3:** LncRNAs that were predicted as miRNA targets and eTMs in melon

LncRNA ID	miRNA ID	miRNA sequence	LncRNA sequence
Targets			
LNC_012115	Cm-miR156	ACTGTCTTCTATCTCTCGTG	TGACAGAAGAGAGTGAGCAC
LNC_011287	Cm-miR156	TTCTGTCTTCTATCTCTGTT	TTGACAGAAGATAGAGAGCAC
LNC_011878	Cm-miR156	CACTGTCTTCTCTCACTCGTG	TTGACAGAAGATAGAGAGCAC
LNC_012070	Cm-miR156	AACCGTCTTCTGTTTCTCGTT	TTGACAGAAGATAGAGAGCAC
LNC_013946	Cm-miR156	AACTGTCTTCTCTCACTCGTG	TTGACAGAAGATAGAGAGCAC
LNC_021671	Cm-miR156	ACTTTCTTCTATTTTTCGTT	TGACAGAAGATAGAGAGCAC
LNC_013934	Cm-miR159	AAACTTATCTTCCCTCGAGT	TTTGGATTGAAGGGAGCTCTA
LNC_027695	Cm-miR159	TAACCTGACTTCCCTCGAGGA	TTTGGATTGAAGGGAGCTCTA
LNC_006537	Cm-miR162	AGCTATTTCGATACGTAGTC	TCGATAAACCTCTGCATCCAG
LNC_016936	Cm-miR164	ACCTCTACGTCTCGTGTACTC	TGGAGAGGCAGGGCACATGCT
LNC_017766	Cm-miR164	ACTTCTCCGTCCCGTATTCGA	TGGAGAGGCAGGGCACATGCT
LNC_020709	Cm-miR164	ACTTCTCCGTTACGTGTAGA	TGGAGAGGCAGGGCACATGCT
LNC_000218	Cm-miR166	AGCCTGGTCCGAAGTAAGGGG	TCGGACCAGGCTTCATTCCCC
LNC_008758	Cm-miR166	AGCCTGGTCCGAAGTAAGGAG	TCGGACCAGGCTTCATTCCCC
LNC_018819	Cm-miR167	AGTTCGACGGTCGTACTAGAT	TGAAGCTGCCAGCATGATCTA
LNC_021730	Cm-miR167	GCTTCGACTGTCGTACTGTT	TGAAGCTGCCAGCATGATCTA
LNC_022241	Cm-miR168	TCGCTTGGTGCAGGTCGGGA	AGCGAACCACGTCCAGCCCT
LNC_004566	Cm-miR169	TTCGTTTCCTACTTAACCAAC	AAGCCAAGGATGAATTGCCGG
LNC_010303	Cm-miR169	ATCGGTTCCTGCTGAACGGCG	AAGCCAAGGATGAATTGCCGG
LNC_022185	Cm-miR1863	AGCTCTGATACCATGTTAGATTTG	ACGAGACTATGGTATAATTTGAC
LNC_027695	Cm-miR319	TTGGACTGAAGGGAGCTCCC	AACCTGACTTCCCTCGAGGA
LNC_008308	Cm-miR390	AAGCTCAGGAGGGATAGCGCC	GTCGAGTTCTCCCTATCTGT
LNC_005952	Cm-miR393	TCCAAAGGGATCGCATTGATC	AGGGTTACCTAGCGTAACTAC
LNC_014927	Cm-miR394	TTGGCATTCTGTCCACCTCC	AACCGTAAGACAGGTGGAGG
LNC_024182	Cm-miR395	CTGAAGTGTTTGGGGGAACTC	TACTTCACAAACTCCCTTAT
LNC_006804	Cm-miR398	TGTGTTCTCAGGTCGCCCCTG	ACACAAGAGTCCAGTGGGGAA
LNC_014183	Cm-miR398	TGTGTTCTCAGGTCGCCCCTG	CCATAAGAGTCCGGCGG-AAC
LNC_020691	Cm-miR399	TGCCAAAGGAGAATTGCAC	TCTGTTTCCTCTTAACGG
LNC_022982	Cm-miR399	TGCCAAAGGAGAATTGCAC	ACAGTTTCCTCTTTACGTA
LNC_005545	Cm-miR477	CTCTCCCTCAAAGGCTTCTG	GGGTGGGAGTTTCCGAAAG
LNC_007655	Cm-miR530	TGCATTTGCACCTGCACCTT	ATGTAAACGTGGATGTAGACA
LNC_004049	Cm-miR854	GATGAGGATAGTGAGGAGGAG	CTACTCCTATCACTCCTCCTC
LNC_012258	Cm-miR854	GATGAGGATAGTGAGGAGGAG	CTACCCCTATCACTACTCCTC
LNC_020802	Cm-miR854	GATGAGGATAGTGAGGAGGAG	TCAGTCCTATCACTCCTTCTC
LNC_026553	Cm-miR854	GATGAGGATAGTGAGGAGGAG	CTTCTCCTATCACTCCTCCTC
LNC_011348	Cm-miR858	TCTCGTTGTCTGTTCGACCTT	AGGGCAACAGGCAAGCTTGTT
LNC_007955	Cm-miR7129	AGTCAAATCTAAACGATCGTGTAT	ACAGTTTAGATTTGCTAGCAAATA
LNC_008970	Cm-miR7129	AGTCAAATCTAAACGATCGTGTAT	TAAGTTTAGATTTGCTAGCACATG
LNC_009809	Cm-miR7129	AGTCAAATCTAAACGATCGTGTAT	TTGGTTTAGATTTGCTGACACATG
LNC_010638	Cm-miR7129	AGTCAAATCTAAACGATCGTGTAT	CGGTTTAGATTTGCTAGAACATT
LNC_016701	Cm-miR7129	AGTCAAATCTAAACGATCGTGTAT	TTAGTTTAGATTTGCTAGCACATG
LNC_023506	Cm-miR7129	AGTCAAATCTAAACGATCGTGTAT	TCAGTTTAGATTTGTTAGCACATG
LNC_024789	Cm-miR7129	AGTCAAATCTAAACGATCGTGTAT	GAAGTTTAGATTTGCTAGCACGA
LNC_021385	Cm-miR7130	GTTTGGAATGTGCGAGATGTGTGC	TCAACTTTACACGCTCTACACACG
eTMs			
LNC_015061	Cm-miR156	TGACAGAAGA---GAGTGAGCAC	ACTGTCTTCTAAGTTCACACGTG
LNC_001794	Cm-miR159	ATTGGATTGA---AGGGAGCTCCT	TAACCTAACTATGTTCCTCGAGTA
LNC_005497	Cm-miR167a	TGAAGCTGCC---AGCATGATCTT	ACTTCGACGGCTTTTGTACAAGGA
LNC_004707	Cm-miR167b	TGAAGCTGC---CAGCATGATCTG	ACTTCGACGACTGTCGTGATAGGC
LNC_001168	Cm-miR169	GAGCCAAGAA---TGACTTGCCGG	CTCGGTTCTTGAAACTGAACGGCT
LNC_027362	Cm-miR172	AGAATCTTGA---TGATGCTGCAT	TATTAGAACTACTACTATGACGCA
LNC_017301	Cm-miR394	TTGGCATTC---TGTCCACCTCC	AACCGTAAGTCCATAGGTGAAGT
LNC_013466	Cm-miR395	TTGAAGTGTT---TGGGGGAACTC	AACTTCACAAAACACTCACTTGAG
LNC_005015	Cm-miR398	TGTGTTCTC---AGGTCACCCCTT	ACACAAGAGAACTCCAGTCGAGAA
LNC_019732	Cm-miR477	CTCTCCCTC---AAAGGCTTCTG	GAGAGGGAGAGGTTTTCTAAAAC
LNC_023016	Cm-miR854	GATGAGGATA---GTGAGGAGGAG	CTACTCCTATTGCCACTCTTACTA
LNC_027169	Cm-miR854	GATGAGGATA---GTGAGGAGGAG	CTACTCCTACGCTCACTACTACTT
LNC_027417	Cm-miR854	GATGAGGATA---GTGAGGAGGAG	CTACTCCTATTAAAAGTCCTGCTC

## Discussion

Emerging evidences showed that lncRNAs play significant roles in multiple biological processes, such as plant growth and development, fruit ripening, drought and salt stress response [13, 25]. In particular, lncRNAs have also been shown to be involved in the resistance against multiple diseases in several plants [26, 27]. However, there is limited information about the roles of lncRNAs and their regulatory mechanism in resisting to PM infection in plants. Recently, the availability of melon genome sequences provided reference information for non-coding regions annotation and their functional analysis. In the present study, high-throughput RNA-seq and comparative transcriptome analysis were performed to identify PM-responsive lncRNAs from the leaves of PM-resistant and PM-susceptible melons. In total, 11,612 lncRNAs were finally discovered. The number of hc-lncRNAs in melon was higher than that in *Arabidopsis* (6480), rice (2965), tomato (3679) and Chinese cabbage (4594), which can be attributed to the number of samples used for sequencing in our study were larger than that of other species. Furthermore, it was observed that lncRNAs in melon were relatively shorter in length and contained fewer exons compare to protein-coding transcripts, which was consistent with the results from all other plants [8, 11].

Differential expression analysis revealed that a large number of lncRNAs were significantly differentially expressed after PM infection in both PM-resistant and PM-susceptible melons, which clearly suggested that these PM-responsive lncRNAs might be important regulators in PM resistance. A total of 183 and 387 lncRNAs were specifically differentially expressed in PM-susceptible melon and PM-resistant melon, respectively. More importantly, qRT-PCR analysis confirmed that the fold change of various lncRNAs in PM-resistant melon were larger than that in PM-susceptible melon, implying that a distinct disease response and function might exist in both melons. Thus, further functional study will be focused on these 387 DELs that were likely to play significant roles in resisting to PM, which contribute to the resistance phenotype in PM-resistant melon. Among 387 DELs, many lncRNAs such as LNC_019333, LNC_018800, LNC_023803, LNC_018062, LNC_010059 were obviously induced at both 24 and 48 hpi compared to mock samples after PM infection in PM-resistant melon, whereas these lncRNAs showed no differential expression either at 24 or at 48 hpi in PM-susceptible melon, which further confirmed that these lncRNAs might play vital roles in the biological processes that against to PM infection.

Previous studies have reported that the interaction between lncRNAs and their targeted mRNAs was one of the most important functional patterns of lncRNAs, and lncRNAs mainly function in regulating the expression of their neighboring genes either in cis or in trans manners [6, 28]. To understand the regulatory pathways of lncRNAs in gene expression during PM infection, potential target genes of the DELs were predicted. Functional annotation results showed that a large number of target genes of lncRNAs encoded proteins that were involved in redox processes such as cytochrome P450, glutathione reductase and peroxidase. It has been proposed that glutathione reductase plays important roles in ROS scavenging pathway to prevent oxidative damage, which alleviates cell membrane injury after pathogen infection. Recently, it has been demonstrated that overexpression of glutathione reductase gene (*SlGRE21*) in tomato reduced ROS accumulation and enhanced the resistance against *P. infestans* [29]. Meanwhile, eight target genes, encoding for disease resistance protein, were identified including pathogen-related gene, LRR receptor gene, and universal stress gene. Moreover, five genes encoding for transcription factors, such as WRKY, ERF, MYB and NAC, were also discovered. In the past decades, increasing studies have revealed that a large number of WRKY family transcription factors were involved in response to biotic defense (bacterial, fungal and viral pathogens) [30]. Subsequently, our qRT-PCR results verified that similar expression patterns were induced between LNC_018062 and *MELO3C015771* (glutathione reductase coding gene), LNC_018800 and *CmWRKY21* after PM infection, suggesting that lncRNAs might control the expression of *CmWRKY21* and redox pathway genes, leading to the resistance to PM in melon.

Interestingly, a *MLO* family gene that acts as susceptibility factor towards PM was found to be targeted by LNC_014937. It is speculated that plant-specific MLO proteins contain seven transmembrane domains, which are likely to modulate vesicle-associated defense responses at the cell periphery [31]. MLO resistance has been reported in barley, *Arabidopsis*, pea, cucumber, tomato and many other species. In tomato, the loss-of-function of *MLO* family gene (*SlMLO1*) led to high resistance against PM [32]. Besides, in *Arabidopsis thaliana*, T-DNA insertion mutations in three *MLO* homologs (*AtMLO2*, *AtMLO6* and *AtMLO12*) contributed to significant PM resistance, although the mutation in *AtMLO2* had a significant effect compared to mutations in other two genes [33]. These studies showed that MLO proteins in plants function as characteristic susceptibility genes (S-genes) and play a negative regulatory role in resisting to PM. In the present study, qRT-PCR results revealed that the expression level of both LNC_014937 and *CmMLO5* in PM-resistant melon was significantly higher than that in PM-susceptible melon. Meanwhile, the expression of both LNC_014937 and *CmMLO5* was significantly down-regulated in PM-resistant melon, although no differential expression was found in PM-susceptible melon, implying that the LNC_014937 and *CmMLO5* module might play important roles in melon responses to PM. Subsequently, further molecular and biological experiments should be carried out to elucidate their biological function and regulatory mechanisms in resisting PM infection.

It has been reported that lncRNAs can control gene expression through various pathways. They can act as targets or eTMs of miRNA to restrict the cleavage of target mRNA mediated by miRNA, and thus activate target gene expression [24, 34]. Similar to the results in *Arabidopsis*, rice, cotton and other plants, many melon lncRNAs were predicted to be miRNAs target or decoys. In the present study, 56 lncRNAs were predicted to be potential targets or eTMs of 32 family miRNAs including miR156, miR159, miR164, miR166, miR167, miR169 and others. Among them, several miRNAs, such as miR398, miR477, miR854 and miR858, have been proved to play important roles in response to various biotic and abiotic stresses. Recently, miR398 was found to be involved in immunity against the blast fungus through regulating two genes encoding Cu/Zn-superoxidase dismutase (*CSD*) [35]. Besides, miR858-mediated regulation of phenylpropanoid biosynthetic pathway also played important role in *Arabidopsis* immunity [36]. These results demonstrated that certain interactions between lncRNAs and miRNAs may exist in melon, which provides a solid foundation for further investigation in the function of lncRNAs in PM tolerance.

## Conclusions

In this study, a total of 11,612 hc-lncRNAs were identified in melon. Further characterization analysis showed that lncRNAs in melon were distributed across all 12 melon chromosomes, and > 85% were from intergenic regions. Besides, lncRNAs in melon were relatively shorter in length and contained fewer exons compare to protein-coding genes. A total of 407 and 611 lncRNAs were found to be differentially expressed after PM infection in PM-susceptible and PM-resistant melons, respectively. Furthermore, 1232 putative targets of differentially expressed lncRNAs were discovered and functional annotation showed that a large number of target genes of lncRNAs encoded proteins that were involved in redox processes, such as cytochrome P450, glutathione reductase and peroxidase. Interestingly, a number of lncRNAs can act as potential miRNA precursors. Meanwhile, lncRNAs could also act as targets or eTMs of miRNAs. Collectively, our findings provide new insights into the role of lncRNAs for further study on the function and regulatory mechanisms of lncRNAs in PM resistance.

## Methods

### Plant materials, growth conditions and powdery mildew fungus inoculation

A highly resistant cultivated melon (M1) and a highly susceptible cultivated melon (B29) to powdery mildew fungus were selected as plant materials and grown in a greenhouse with a photoperiod of 16/8 h (day/night) at 28 °C/20 °C (day/night). M1 is an inbred line that was self-pollinated for thirteen generations, with thick rind and high net density. B29 is an inbred line separated from a commercial cultivar, with a thin and smooth rind (no netting). The genetic background of these two lines has been highly stable. Powdery mildew fungus was collected from cultivated melon grown in the experimental farm of Shandong Academy of Agricultural Sciences with normal day/night period. Plants with two or three true leaves were inoculated by powdery mildew fungus at a concentration of 1 × 10^6^/mL as previously described [37]. Control samples were treated with water (mock). Leaves of both M1 and B29 were harvested at 24 and 48 h post inoculation (named as M24, M48, B24, B48, respectively), and immediately frozen in liquid nitrogen and stored at − 80 °C for the following RNA extraction. Three biological replicates were prepared for each sample.

### Total RNA extraction, library construction and paired-end strand-specific sequencing

Total RNAs were extracted from all samples using Trizol reagent following the manufacturer’s instructions (Invitrogen, CA, USA) and the integrity was examined on 1% agarose gel. RNA concentration and quality were measured by NanoPhotometer spectrophotometer (IMPLEN, CA, USA) and Bioanalyzer 2100 system (Agilent Technologies, CA, USA). A total amount of 3 μg RNA per sample was used as input material for RNA sample preparation. Firstly, ribosomal RNA was removed using rRNA Removal Kit (Epicentre, USA), and rRNA free residue was purified by ethanol precipitation. Subsequently, sequencing libraries were generated using rRNA-depleted RNA by NEBNext Ultr Directional RNA Library Prep Kit for Illumina (NEB, USA) following manufacturer’s instructions. After library generation, 150 bp paired-end reads were generated on an Illumina Hiseq 4000 platform. The raw sequence reads are available for download from the NCBI sequence read archive database (Accession number: SRR9129105-SRR9129122).

### RNA transcripts assemble and identification of lncRNAs

To assemble full-length transcripts, the raw data were preprocessed by the Fastx-toolkit pipeline (http://hannonlab.cshl.edu/fastx_toolkit/) to trim the adapter sequences and remove low-quality sequences. All clean reads were aligned to melon reference genome (http://cucurbitgenomics.org/organism/18) using HISAT2 (v2.0.4; https://ccb.jhu.edu/software/hisat2/index.shtml). Only reads with no more than two mismatches were used to generate full-length transcripts of each sample separately using StringTie (version 1.3.1; http://ccb.jhu.edu/software/stringtie/). To identify lncRNA, all transcripts were firstly aligned to housekeeping ncRNA databases (version 1.2; http://bioinf.scri.sari.ac.uk/cgi-bin/plant_snorna/home) to exclude tRNAs, snRNAs and snoRNAs. Then, the remaining transcripts were compared with melon genome annotated protein sequences (http://cucurbitgenomics.org/organism/18) using BlastX. Non-redundant transcripts with significant alignment (*P* < 1.0E-5, identity> 90%, coverage> 80%) to melon proteins were excluded. Perl scripts were used to exclude transcripts shorter than 200 nucleotides and transcripts with a FPKM score higher than 1 in at least one sample. Finally, the remaining transcripts were uploaded to the Coding Potential Calculator (CPC), Coding-Non-Coding-Index (CNCI), Pfam Scan (Pfam-sca) and phylogenetic codon substitution frequency (phyloCSF) programs to test protein-coding potential. Transcripts predicted with coding potential by at least one of the four tools above were filtered out, and those without coding potential were identified as candidate lncRNAs.

### Quantification and differential expression analysis of lncRNAs

Cuffdiff program (v2.1.1) was used to calculate FPKM values of lncRNAs in each sample [38]. FPKM means fragments per kilo-base of exon per million fragments mapped, which is calculated based on the length of the fragments and reads count mapped to this fragment. Furthermore, Cuffdiff program (v2.1.1) was also used to calculate adjusted *P*-values between two samples with three biological replicates. Then, differentially expressed lncRNAs were identified using a criterion of |log_2_ fold change| ≥ 1 and adjusted *P*-values < 0.01. The adjusted *P*-values were calculated using Benjamini-Hochberg procedure. Hierarchical clustering heat map was generated using pheatmap in R package (https://www.r-project.org/) according to the euclidean distance method.

### Validation of differentially expressed lncRNAs by qRT-PCR

To confirm the differential expression of lncRNAs, qRT-PCR method was performed to assess the relative expression quantity of lncRNAs. Total RNAs were extracted from the leaf samples using Trizol reagent following the manufacturer’s instructions (Invitrogen, CA, USA). First strand cDNA was synthesized from 3 μg of RNA using random hexamer primer and M-MuLV Reverse Transcriptase. Second strand cDNA synthesis was performed using DNA Polymerase I and RNase H. SYBR Green Master Mix (Bio-Rad, Hercules, California) was used in all qRT-PCR reactions with an initial denaturing step of 95 °C for 5 min, followed by 40 cycles of 95 °C for 20 s, 65 °C for 20 s and 72 °C for 20 s on an ABI 7500 Real Time PCR system (Applied Biosystems, Waltham, Massachusetts). Three biological replicates were prepared for each sample and *Cmactin* was used as internal reference gene. Relative expression was calculated using the 2^-△△Ct^ method and all data were expressed as means ± SDs from three independent experiments. Duncan’s multiple range tests were used to determine the statistical difference between samples (*P* < 0.01). Primers used in all qRT-PCR experiments are listed in Additional file 4: Table S2.

### Identification of lncRNA targets and gene ontology enrichment analysis

To identify the target genes of differentially expressed lncRNAs, a Perl script was used to identify cis target genes located 10 kb upstream or downstream of lncRNAs, and for the identification of antisense target genes that can interact with lncRNAs to produce RNA duplex, RNAplex tool was used to examine the RNA duplex formation by calculating minimum free energy (MFE) based on their respective structures [39]. Gene Ontology (GO) enrichment analysis of lncRNA target genes was performed using GOseq R package [40]. GO terms with corrected *P*-value < 0.05 were considered significantly enriched. We used the Benjamini-Hochberg Correction for the *p*-value to obtain a corrected *p*-value.

### Identification of lncRNAs that act as miRNA precursors, targets or eTMs

LncRNAs that may act as miRNAs precursors were predicted by aligning all lncRNA sequences against the known miRNA precursor sequences in miRBase database using BLASTN with an identity > 90% and cutoff E-value < 1.0E-5. To obtain lincRNAs that can act as miRNA targets, psRNATarget was used with following rules: at most, one mismatch was allowed between the 9th and 12th positions of the 5′-end of miRNA sequences, the total number of mismatches in other regions were not allowed to exceed 4, and no continuous mismatches were allowed [41]. The miRNA eTMs from all lncRNAs were predicted using psMimic software according to following rules: (1) bulges were only permitted at 5′-end ninth to 12 th positions of miRNA sequence; (2) the bulge in lncRNAs should be composed of only three nucleotides; and (3) total mismatches within lncRNA and miRNA pairing regions should be no more than three except for the central bulge [42].

## Supplementary information

**Additional file 1: Table S1.** Summary statistics of RNA-seq data and mapping result.

**Additional file 2: Figure S1.** The correlation coefficients among all 18 samples.

**Additional file 3: Figure S2.** The most enriched GO terms of lncRNA targets in biological process.

**Additional file 4: Table S2.** Oligonucleotide primer sequences used for qRT-PCR.

## Data Availability

The raw sequence reads are available for download from the NCBI sequence read archive database (Accession number: SRR9129105-SRR9129122).

## Abbreviations

DEL: Differently expressed lncRNA
eTM: Endogenous target mimic
FPKM: Fragments per kilo-base of exon per million fragments
GO: Gene ontology
lncRNAs: Long non-coding RNAs
MFE: Minimum free energy
miRNA: microRNA
MLO: Mildew Locus O
PM: Powdery mildew
qRT-PCR: quantitative RT-PCR
siRNA: Small interfering RNA
