# Supplementary material for: Comparative transcriptome analysis uncovers regulatory roles of long non-coding RNAs involved in resistance to powdery mildew in melon
Source: BMC Genomics. 2020 Feb 5;21:125. doi: 10.1186/s12864-020-6546-8 (PMC7003419; doi:10.1186/s12864-020-6546-8)
Supplement: Supplementary file 1 — Additional file 1: Table S1. Summary statistics of RNA-seq data and mapping result. [file 12864_2020_6546_MOESM1_ESM.docx]

**Table S2 Summary statistics of RNA-seq data and mapping results.**

| Sample name | Raw reads | Clean reads | Clean bases | Total mapped unique reads(%) |
| --- | --- | --- | --- | --- |
| M0_1 | 108010586 | 100535446 | 15.08G | 84.90 |
| M0_2 | 101356468 | 95155226 | 14.27G | 85.91 |
| M0_3 | 106911056 | 99862912 | 14.98G | 84.73 |
| M24_1 | 105996486 | 98223418 | 14.73G | 85.83 |
| M24_2 | 105865820 | 98135728 | 14.72G | 85.68 |
| M24_3 | 102783160 | 95676696 | 14.35G | 85.81 |
| M48_1 | 114601256 | 106591602 | 15.99G | 85.97 |
| M48_2 | 105301178 | 97652272 | 14.65G | 83.95 |
| M48_3 | 108250146 | 106440110 | 15.97G | 82.68 |
| B0_1 | 117713532 | 115800212 | 17.37G | 83.15 |
| B0_2 | 108293262 | 106406988 | 15.96G | 84.32 |
| B0_3 | 123308482 | 120942420 | 18.14G | 84.64 |
| B24_1 | 100734002 | 99074280 | 14.86G | 85.50 |
| B24_2 | 110841694 | 109081240 | 16.36G | 84.35 |
| B24_3 | 121153390 | 119067176 | 17.86G | 84.47 |
| B48_1 | 116168260 | 114314170 | 17.15G | 84.10 |
| B48_2 | 125834776 | 107675836 | 16.15G | 84.28 |
| B48_3 | 110926652 | 94267112 | 14.14G | 85.00 |
