# Supplementary figures and images for: Comparative transcriptome analysis uncovers regulatory roles of long non-coding RNAs involved in resistance to powdery mildew in melon
Source: BMC Genomics. 2020 Feb 5;21:125. doi: 10.1186/s12864-020-6546-8 (PMC7003419; doi:10.1186/s12864-020-6546-8)

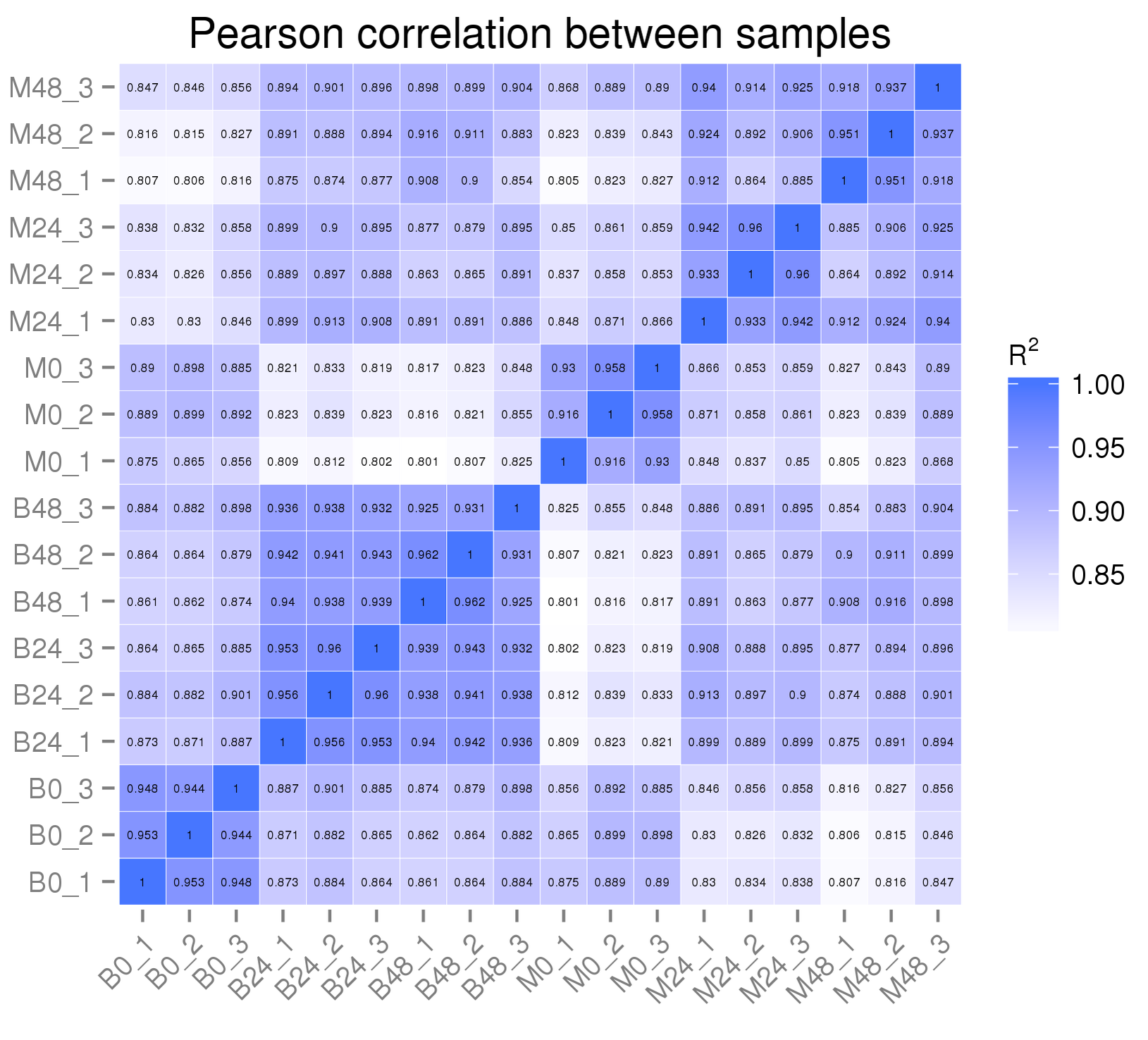

Supplement: Supplementary file 2 — Additional file 2: Figure S1. The correlation coefficients among all 18 samples. [file 12864_2020_6546_MOESM2_ESM.tiff]

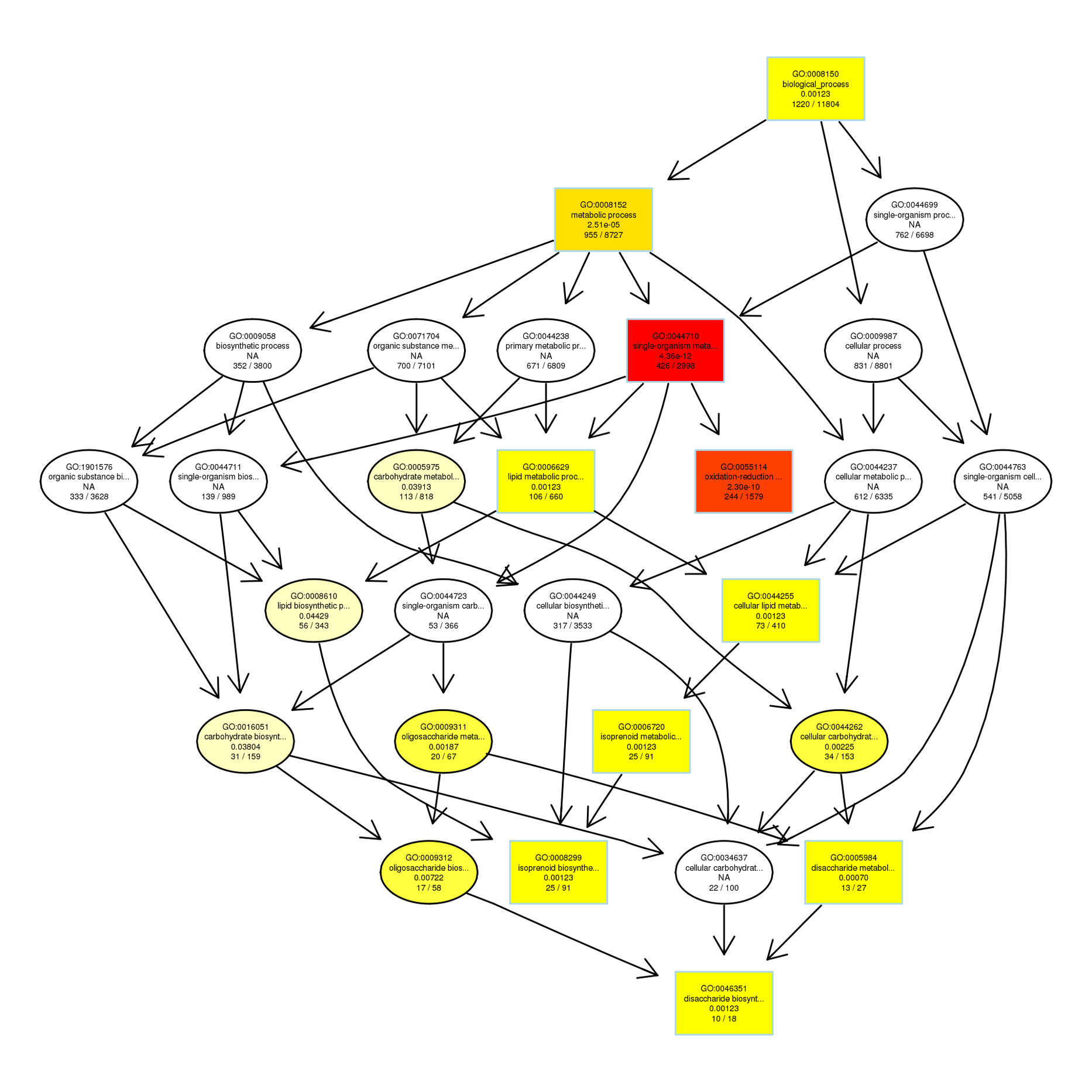

Supplement: Supplementary file 3 — Additional file 3: Figure S2. The most enriched GO terms of lncRNA targets in biological process. [file 12864_2020_6546_MOESM3_ESM.tiff]
